# Comparative analysis of public and expert perceptions of electrified vehicles in the European Union

**DOI:** 10.1038/s41598-025-06071-0

**Published:** 2025-07-01

**Authors:** Andromachi Mourtzouchou, Andres L. Marin, Lorenzo Laveneziana, Alessandro Tansini, Jaime Suarez, Ada Garus, Monica Grosso, Fernando Martínez-Plumed, Rubén Cordera, Georgios Fontaras, Biagio Ciuffo

**Affiliations:** 1 Departamento de Transportes y Tecnología de Proyectos y Procesos, Universidad de Cantabria, Santander, Spain; 2 VRAIN, Universitat Politècnica de València, Valencia, Spain; 3 Department of Energy, Politecnico di Torino, Turin, Italy; 4 Joint Research Centre, Ispra, Italy; 5 Departamento de Geografía, Urbanismo y Ordenación del Territorio, Universidad de Cantabria, Santander, Spain

**Keywords:** Mechanical engineering, Psychology and behaviour, Environmental impact

## Abstract

The transition to electric vehicles is widely regarded as an effective measure to combat climate change, and its success will utterly depend on the willingness of consumers to shift to this technology, making it essential to examine public perception when designing supportive policies. Nonetheless, public perception can be biased by multiple factors such as fake news, prejudices and misgivings, leading to a significant divergence from the views of experts in the transportation field. This paper examines the differences between the views of these two groups on electric vehicles, with a specific focus on the current perception in the European Union. The research adopts a two-fold approach, starting with an empirical driving study, followed by a series of focus group discussions with transportation experts and an analysis of Twitter (formally X) data. The data for this study was collected over five years, from June 2019 to June 2024. The findings indicate that both groups generally maintain a neutral stance, with neutrality appearing in 50% of their responses. However, experts tend to prefer battery electric vehicles, positively influenced by their driving experiences. In 40% of their comments, they report favourable sentiments toward these vehicles. Conversely, plug-in hybrid electric vehicles are favoured by the general public, with 35% of tweets indicating a positive attitude, as they are viewed as a more established technology. Differing perspectives on vehicle charging infrastructure are evident: specialists regard it as a hindrance to widespread adoption, with 30% expressing negative views, whereas the general population appears less concerned and more focused on the feelings and the experience of driving.

## Introduction

In response to the growing apprehensions highlighted by the 6th report of the International Panel on Climate Change (IPCC)^1^, the most recent Conference of the Parties (COP)^2^ has widely acknowledged the necessity for immediate measures to address global warming. The level of international commitment is becoming increasingly evident, as all major world economies, from the United States (USA) to India, have pledged to achieve net zero emissions targets^3^. One sector that historically has been difficult to decarbonise is road transport^4^, where numerous measures and technologies have been proposed yet so far with limited effectiveness^5^. In the EU in particular, which was the first region to adopt mandatory CO_2_ targets for cars, decarbonisation has progressed, but more ambitious actions are needed for achieving the block’s 2035 zero fleet emissions target^6^ that will require extensive electrification^7^.

Road transport decarbonisation is not just a matter of technology deployment^8–10^ , but a transition with fundamental effects on the economy and society^11^. As such, it requires interventions at multiple levels, like infrastructure, production chains, and consumer acceptance and use adaptation. This last point is crucial for the uptake of any novel technology solution. In particular, electrified vehicles are sensitive to user perception for multiple reasons, as highlighted in recent literature^12,13^. In addition to any regulatory measures and standards, cars’ markets are worldwide extensively influenced by media coverage, advertising, word of mouth, user habits, and widespread social media, which can be potentially unreliable sources of information^14,15^.

The scope of the present study is to explore the alignment between public perception and transportation experts related to the sentiment surrounding vehicle electrification. Previous studies have already highlighted the importance of public acceptance and perception for the rapid introduction of electric vehicles (EVs)^12,13,16–18^. These studies usually employ focus groups (FG), questionnaires, or on-line social networks. Although past studies have conducted various FG analyses, there is a lack of research focused on the examination of transportation experts regarding the shift to vehicle electrification. In terms of public opinion, various studies have been conducted using on-line social media data. However, no research compiles opinions in all official languages of the EU (language rules—European Union: https://european-union.europa.eu/principles-countries-history/languages_en) and countries.

The present study attempts to go beyond the usual approaches and address limitations through a combined approach. To understand the perception of transportation experts towards electric vehicles and the key factors influencing them, the study first relied on targeted vehicle campaigns. For the initial phase of the study, 10 transportation experts who did not already own an electric vehicle volunteered to use three types of vehicle: a traditional internal combustion engine (ICE) vehicle, a plug-in hybrid electric vehicle (PHEV) and a battery electric vehicle (BEV) for periods ranging from 2 to 4 weeks each. Subsequent to the driving sessions, FG discussions were held to gather the opinions of experts on various powertrain technologies related to the three vehicles driven. As a follow-up step, the study relied on data collected from X (formerly known as Twitter) from all EU countries and official languages. Finally, an analysis of sentiment and emotions was conducted in both samples, FG and public opinion. The outcomes of this analysis were compared to assess the alignment with respect to vehicle electrification.

## Literature review

Comparing expert and non-expert perspectives is essential for understanding the relationship between knowledge creation, communication, and application across various fields. This comparison is not limited to a single discipline but is a tool to examine how specialised knowledge is constructed and disseminated to those outside the field. By contrasting these views, researchers can identify discrepancies, knowledge gaps, and communication breakdowns, highlighting factors influencing public attitudes and policy preferences^19–22^. This is particularly valuable in areas such as risk perception and communication, science communication, decision making, design, education, and emerging technologies, where bridging the gap between expert consensus and public understanding is crucial^19,20,22–24^.

Perception of public opinion on BEVs is usually gauged using surveys, questionnaires, and interviews. Although useful, these methods face limitations such as restricted sample sizes, biases, geographic limitations, and challenges in tracking opinion progression over time^13,16^. Recognising these limitations, researchers have increasingly turned to online social networks such as Twitter and Reddit as rich, timely and scalable data sources for understanding public perceptions of BEVs^12,13,16,25^. These platforms offer vast repositories of user-generated content, including tweets, comments, and interactions, providing valuable insights into real-time public discourse and sentiment^12,13,16,26^.

A study analysing Reddit data^13^ identified cost as the main consumer concern about BEVs and noted increased attention to BEV-related policies, suggesting government incentives influence consumer attitudes towards BEV adoption. Meanwhile, a study incorporating Twitter data^12^ found news media and politicians as key opinion leaders in EV discourse, indicating their role in shaping public opinion. Both studies provide insights essential for stakeholders such as policymakers, car manufacturers, and energy providers to formulate strategies to increase the uptake of BEVs and address consumer fears. At the same time, Balla et al.^16^ studied public opinion progression on BEVs from 2012 to 2022, noting increased awareness and financial considerations as principal obstacles. Their analysis likely encompasses a global geographical range but is limited to English tweets, introducing potential demographic bias.

Various methods have been used to explore the views of specific groups. These methods include conducting FGs^27,28^ and conducting a survey^29,30^. FGs have proven to be an effective method for gaining insight into public views and opinions within transportation studies. They provide a qualitative approach for thoroughly examining complex topics and new technologies, such as BEVs^17,18,31–33^ and future developments such as autonomous vehicles (AVs)^27,34^.

In e-mobility, FGs have investigated public perceptions of BEVs and gathered expert information. For example, Kester et al.^31^ distinguished BEV owner and non-owner views in Nordic cities. Recently, Ramesan et al.^18^ used FGs with experts like auto industry professionals and policymakers to explore EV adoption challenges. Similarly, Silva et al.^17^ held FGs in six Midwestern US cities, exploring perspectives on EV shifts from auto workers and managers, revealing opinion divergence. Other studies have used FGs for specific e-mobility aspects. Other studies have used FGs to explore specific aspects of e-mobility^32,33^. The study by Sovacool et al.^32^ explored gender aspects of e-mobility and EV preferences using FG and a variety of qualitative and quantitative methods. Noel et al.^33^ used a mixed methods approach including FGs, to explore range anxiety.

Despite the growing body of research on e-mobility in different countries^25,35,36^, there is a lack of focus on electric transportation perceptions in EU countries and studies on expert perspective on BEVs. Although literature often juxtaposes expert and public opinions, research on the transition to electrified vehicles comparing these perspectives is scarce.

## Results

A sentiment analysis was conducted by integrating a targeted vehicle campaign aimed at transportation specialists with Twitter data aggregation, allowing an evaluation of the alignment between these two groups. First, a detailed analysis of the FG responses is provided, beginning with an overview of the general assessments of the three vehicle types, followed by a closer examination of specific issues such as the charging experience and shifts in opinions toward EVs.

### Analysis of the focus group data

This section presents both a quantitative and qualitative assessment of the FG discussions, showcasing the thorough evaluations, preferences, and apprehensions of participants concerning the three vehicle types. The discussed topics encompass general driving experiences, particular challenges related to the charging infrastructure, and the collective sentiment and emotional reactions provoked by the vehicles.

#### General evaluation of the three cars

**Fig. 1:**
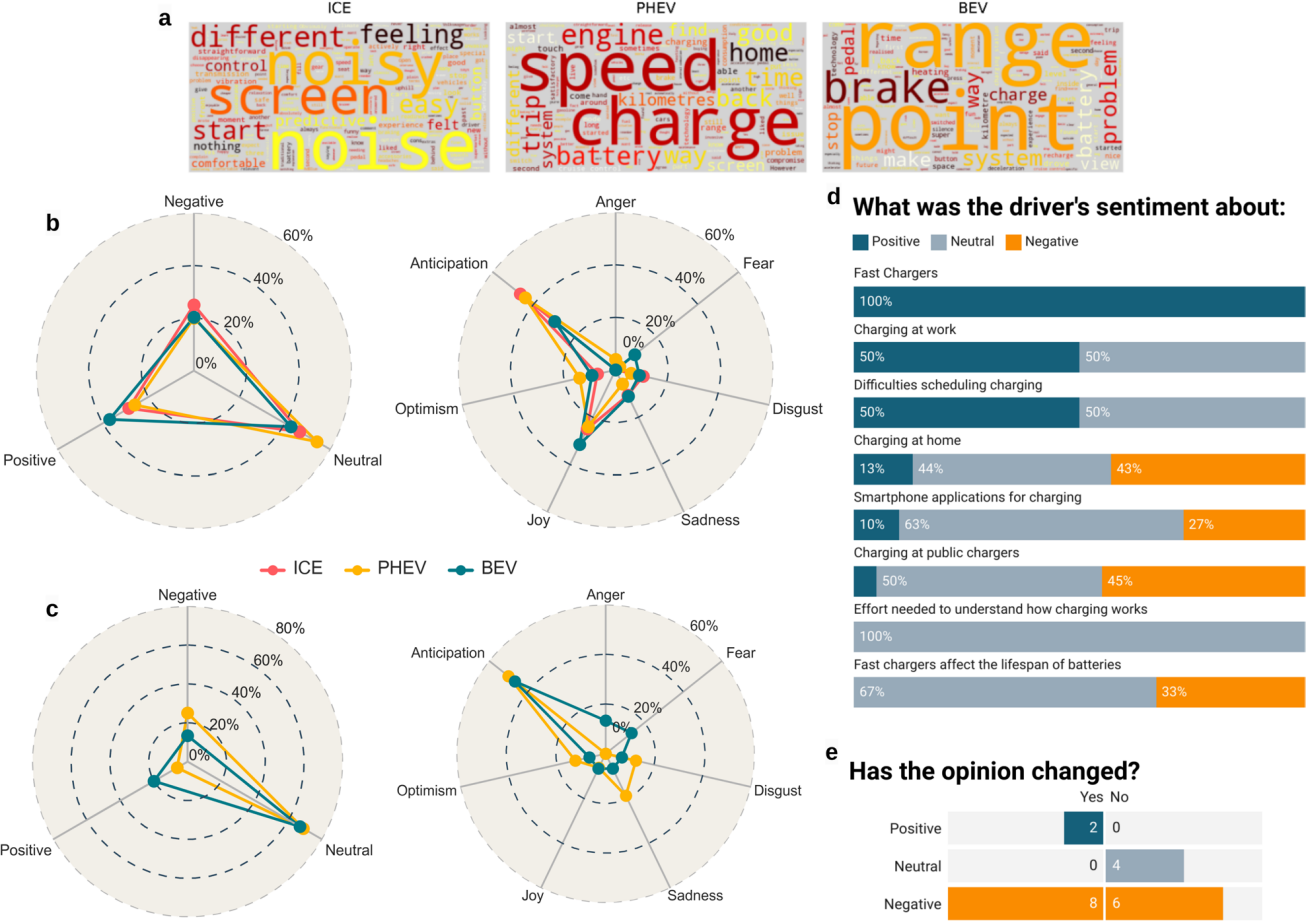
General Evaluation of vehicles by the FG. (**a**) Frequency of words in the *General Evaluation* category for the three cars. (**b**) Sentiment and emotion distribution in the *General Evaluation*. The radial axes depict the percentage distribution of sentiments and emotions per car. (**c**) Sentiment and emotion distribution in the *Charging Experience* discussion. The radial axes indicate the intensity of sentiments and emotions as a percentage. (**d**) Sentiment proportions across different charging topics. This chart visualises the detected sentiments in the most discussed charging-related subtopics of the FGs, providing insights into factors that contribute to the overall evaluation of the charging experience. (**e**) Counts of excerpts in favour (Yes) and against (No) a change in opinion towards EVs, with excerpts coloured to reflect the sentiment detected in drivers’ statements about their opinion change.

In the *general evaluation* phase, the drivers were asked to provide an overall evaluation of the experience with the three cars. The word clouds shown in Fig. 1a offer an overview of the main issues discussed for each car. To maintain clarity and relevance, stop words and non-essential terms were excluded, ensuring that the word clouds accurately represented the frequency of the most important topics.

The discussion of PHEVs and BEVs focused on aspects of driving experience and issues related to charging. As depicted in Fig. 1a, the charging frequency emerged as a prevalent topic in discussions about PHEVs, whereas the driving range was the most frequently mentioned topic in discussions about BEVs. In contrast, drivers perceived ICE vehicles to offer a comfortable driving experience. However, noise and vibration were identified as notable drawbacks of ICE vehicles compared to PHEVs and BEVs (Fig. 1a). Additionally, some participants noted that the ICE vehicles lacked some excitement, which diminished overall enjoyment of the driving experience. A group of users also mentioned that routine maintenance tasks, such as refilling diesel exhaust fluid for pollutants after treatment, posed a slight inconvenience.

The participants characterised the PHEV as more exciting than the ICE, primarily because of its electric propulsion. Nevertheless, some participants expressed concerns about the limited electric range and unpredictability of the PHEV when transitioning between pure electric and fuel-assisted modes.

Additionally, safety issues were highlighted related to Advanced Driver Assistance Systems (ADAS). Because of the vehicle adjusting its speed too harshly depending on the road’s limits, participants perceived this feature as a potential risk.

The BEV was praised for its quiet and comfortable ride, without engine noise or vibrations, along with its responsive power. However, participants pointed out the need to adapt to its rapid acceleration and reported erratic acceleration and braking behaviour, particularly with fluctuating battery charge levels. These aspects were considered potential safety concerns. Additional concerns were raised about the dependency on the charging infrastructure and range anxiety. The access to charging points and the range of the vehicle can differ depending on the area, potentially leading to anxiety during long journeys. Furthermore, the use of air conditioning might affect the driving range.

Safety opinions on the BEV were mixed; while some praised its driver assistance systems, others noted concerns about inconsistent braking, requiring continuous adaptation of driver behaviour, and the infotainment screen being distracting, see Fig. 1a. Overall, drivers tended to favour the simplicity of the ICE vehicle over the more technologically advanced BEV, as the new driving assistance systems in the BEV could potentially distract drivers.

### Sentiment analysis

A comprehensive assessment of ICE, PHEV, and BEV vehicles revealed a majority of neutral opinions: 51% for PHEVs, 44% for ICEs, and 41% for BEVs. ICE vehicles garnered the most negative feedback at 25%, while PHEV and BEV received 20%. Positive feedback was most frequent for BEVs at 39%, with ICEs at 31%, and PHEVs at 29% (Fig. 1b). No statistical differences were observed between ICE and PHEV, nor between ICE and BEV. According to Boschloo’s test^37^, the resulting p-values were 0.34 and 0.48, respectively. Given that these p-values exceed our predetermined significance threshold (*α* = 0*.*22), we can conclude that there is evidence supporting the acceptance of the null hypothesis (*H*_0_). In comparing PHEVs and BEVs with the same *α*, we found no significant difference as the p-value is 0.27.

Analysing the emotions, anticipation (associated to interest or vigilance according to^38^) was the most frequent emotion for all vehicles, with ICE and PHEV at roughly 45% and BEV at 30%. ICE responses included 10% disgust (equated to dislike according to^38^), sadness, and optimism, and with 25% joy. PHEV had 5% anger, 7% disgust, 4% fear, 14% optimism, and 25% joy. BEV elicited the most joy at 31%, the highest fear at 10%, 8% each for disgust and sadness, and 13% optimism. Although there are apprehensions regarding BEVs, there is generally considerable interest and optimism surrounding EVs overall.

#### The charging experience

Participants enjoyed driving the BEV but expressed concerns about the charging infrastructure impacting their overall assess- ment. Range anxiety, induced by the scarcity of charging stations and inconsistent vehicle range, emerged as a crucial issue^39^.

One participant aptly summarised this concern:

*I did not convince friends of mine to go on a trip (from Italy) to Germany with this car (BEV). I wanted to go to Germany, I made some simulations, some plans and found the charging points where we could stop. I told them it was feasible, and we would get there but nobody agreed to take the risk, despite me being an energy engineer who works with electric and plug-in vehicles. Despite showing them the proof that I had done a simulation, and we would get there, and we would be passing through Switzerland where there are thousands of charging stations and powerful ones, I did not manage to convince them.* (Edoardo)

In general, sentiments towards charging were neutral, with PHEVs receiving slightly more negative responses than positive ones. BEVs, however, had a higher percentage of positive than negative responses. The predominant emotion for both cars was anticipation, although negative emotions varied: BEV users felt anger and fear, while PHEV users experienced sadness and disgust. Positive emotions were limited (below 10%), see Fig. 1c. According to the results of Boschloo’s test^37^, it was found that drivers expressed greater satisfaction with the charging experience of BEVs in comparison to PHEVs, as indicated by a p-value of 0.17. The alternative hypothesis (*H*_1_) was framed as *less*, where the contingency matrix was set up such that *p*_2_ represents BEVs and *p*_1_ denotes PHEVs. By accepting *H*_1_, we can infer that *p*_2_ > *p*_1_.

PHEVs received a more severe evaluation compared to BEVs. Two possible reasons are unrelated to the powertrain technology itself: firstly, participants tested PHEVs before BEVs, and noticeable improvements in the charging infrastructure occurred in the interim period (both at the research centre and externally). Secondly, their increasing familiarity with EV charging during the PHEV tests might have influenced their positive judgment of BEVs.

Two primary reasons related to the powertrain also emerged. The first is the limited electric range of PHEVs, as one participant highlighted:

*I found the PHEV was not entirely satisfactory for my needs, and going back and forth from home and work was just sufficient for the range that I had. As soon as I had [inaudible] to do something else the thermal energy started to be switched on. And somehow this would disappoint, because in the end, you say, I have a plug-in hybrid and let us say to go to Milan or somewhere else, I would like to use the battery as much as possible.* (Marco)

Additionally, PHEVs have lower battery capacity, requiring longer charging times and limiting electric-only range^40^.

Many drivers were annoyed with the need to schedule charges and alter habits, though they acknowledged these issues were specific to their situations and not necessarily generalizable^41^. Generally, home and public charging experiences were negatively viewed. Home charging issues included impracticality in urban apartments and the administrative challenges of installing garage chargers. About 90% of sentiments towards home charging were negative or neutral. This negative perception can be attributed to the fact that nearly all participants were not PHEV owners, lacking experience with home charging, which likely influenced their opinions. This is illustrated by the following response:

*I wanted to say for the PHEV, I live in a condominium and I did not have the opportunity to recharge at home, so I went to my father-in-law’s who lives in a standard house 15 kilometres away. I spent one hour taking my car to go home in the evening and in the morning. This is another problem because I think this kind of car is very good for city mobility but if you want to use the car in all situations, it is not acceptable.* (Luca)

Public charging received even more negative feedback due to unreliable chargers, long walking distances from chargers to homes, and lack of features like queue systems and live updates. BEV charging app feedback was mostly neutral to negative, with users citing inaccurate station availability and inconsistent charging fee policies.

In contrast, workplace charging received overwhelmingly positive feedback, appreciated for its convenience during work hours, provided adequate infrastructure was available. Finally, the availability of fast chargers, although not extensively discussed, were positively evaluated for their reduced charging time, though some drivers worried about potentially reduced battery life (Fig. 1d).

Although the findings from these FG sessions might not be directly applicable to every region, whether urban or rural, nor to all European nations, they still provide important insights into the challenges and perceptions related to EV charging. Since the study was carried out in a rural area of Italy, the results mainly pertain to that context, yet they may still be beneficial for broader applications.

#### Changes in opinion

Following the test drives of the three vehicle types (ICE, PHEV, BEV) drivers were asked about any changes in their perspectives regarding the various powertrain technologies. Of the 20 recorded responses, 10 indicated that they did not change their opinion, while 10 acknowledged a change in perspective after comparing the different powertrains (Fig. 1e).

Sentiment analysis revealed predominantly negative feelings, even among those who changed their stance towards BEVs. Based on Boschloo’s test^37^, we identified a positive shift between individuals who modified their opinion and those who remained unchanged. The computed p-value stood at 0.17, supporting hypothesis *H*_1_ which suggests a *greater* effect, given an *α* of 0.27. The contingency table was organized with *p*_1_ denoting opinion change and *p*_2_ none opinion change. Despite a newfound appreciation for EVs, many drivers expressed disappointment due to high purchase costs and inadequate charging infrastructure. Concurrently, it indicates that individuals who were initially more skeptical, due to reliance solely on theoretical knowledge, exhibited a notably more favourable change, especially regarding BEVs. This reinforces the notion that initial skepticism often arises from fear or undervaluation of the unfamiliar, and that firsthand experience can ease these worries.

These barriers prevent an immediate transition to EVs, pushing some to consider hybrids instead. This indicates that while awareness of EVs may rise, it does not necessarily lead to positive sentiments or immediate acceptance. Current policies and incentives seem insufficient to address these barriers, suggesting the need for more targeted measures to support potential EV adopters.

### Comparison of expert and public sentiments

**Fig. 2:**
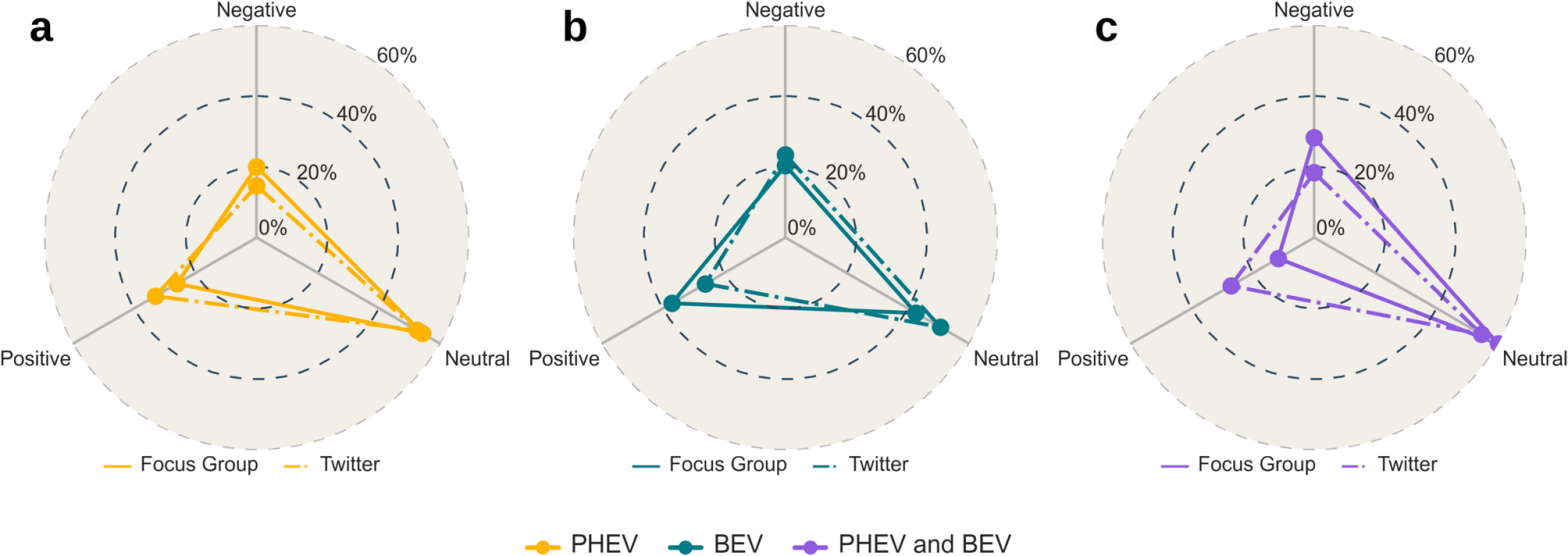
Sentiment comparison between Focus Group and Public Opinion (Twitter), (**a**) Comparison towards PHEVs. (**b**) BEVs, and (**c**) Charging experience. The radial axes in all the figures depict the percentage distributions.

Sentiments from the FG group analysis were compared with Twitter data from EU nations during the driving campaign period (June 2019 to June 2024). This facilitated a contrast between public opinions on Twitter and those expressed by the FG experts. The dominant sentiment in both groups was neutral. However, distinct differences were observed in positive and negative evaluations. For BEVs, Twitter opinions were balanced between positive (20–25%) and negative (20–25%) sentiments. FG experts had a more positive view (around 37%), with 20% negative comments (Fig. 2b). Employing Boschloo’s test^37^, it can be inferred that FG exhibits a more favourable perspective on BEVs compared to Twitter users. The obtained p-value was 0.10. Since this p-value is less than the significance level *α* of 0.22, with *H*_1_ being *less*. The contingency table was constructed, assigning *p*_1_ to the Twitter dataset and *p*_2_ to the FG dataset. FG experts’ positive sentiments likely stemmed from their direct driving experience with BEVs, contrasting with Twitter users’ potential lack of such experience.

Regarding PHEVs (Fig. 2a, FG responses were evenly split between positive and negative (20–25%), while Twitter showed a higher positive sentiment (above 35%) and lower negative sentiment (around 15%). The Boschloo’s test^37^ yielded a p-value of 0.13 with an alternative hypothesis *H*_1_ stated as *grater*. Since this p-value is below the significance level *α* of 0.22, we can accept *H*_1_, suggesting that Twitter users, *p*_1_, show more favourable attitudes toward PHEVs compared to those in the FG, *p*_2_. The Twitter community’s preference for PHEVs might be due to their perceived maturity and independence from an underdeveloped charging infrastructure. In contrast, our FG participants, who were not PHEV owners by choice but rather received them for the study, found the charging more challenging, likely due to their limited experience and lack of preparation.

**Fig. 3:**
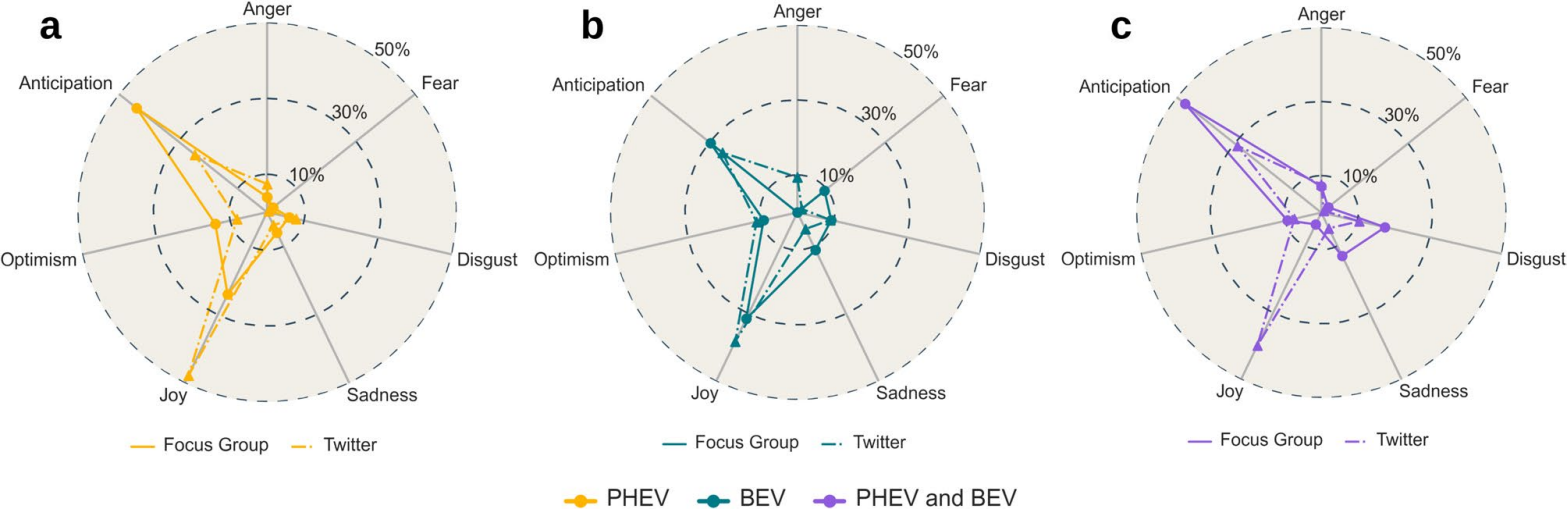
Emotions comparison between Focus Group and Public Opinion (Twitter), (**a**) Comparison towards PHEVs. (**b**) BEVs, and (**c**) Charging experience. The radial axes in all the figures depict the percentage distributions.

For BEVs, both groups showed similar emotions, primarily joy and anticipation (Fig. 3b). Anger was notable in 10% of tweets but nearly absent in FG data (deniting the presence of several Twitter users expressing harsh criticisms towards BEVs). Sadness was found in 10% of FG responses but not in Twitter data, possibly due to direct negative experiences during driving (e.g., from the need to change driving style with respect to a common ICE to anxiety issues of the range). Optimism was moderately expressed: around 10% in both groups.

For PHEVs (Fig. 3a), Twitter users predominantly expressed joy (over 40%), whereas FG showed moderate joy (20%). Rather, the drivers in the FG manifested a neutral interest (anticipation) for the PHEV, about 40% versus 20% of the Twitter users. FG also expressed slightly more optimism (15% vs. 10%). Anger was present in 10% of tweets, but absent in FG discussions. Lastly, sentiments and emotions regarding the EV charging experience were compared. Both groups primarily expressed neutral sentiments (Fig. 2c).

However, FGs showed more negative sentiments (25%) due to poor charging infrastructure, whereas Twitter leaned positively (over 20%). Upon comparison, we acquired a p-value of 0.15 with an alternative hypothesis *H*_1_ specified as *greater*, based on Boschloo’s test^37^. The p-value is less than the chosen *α*, which is 0.27. Statistically, this indicates that Twitter users, denoted as *p*_1_, have a more favourable perception of the charging than the FG, represented by *p*_2_. Regarding this notable difference, it is important to remember that FG drivers expressed different sentiments for the charging experience with the BEV and PHEV. The charging experience for BEVs was rated more positively than for PHEVs. When examining the BEV charging experience alone (Fig. 1c), the sentiment distribution aligns more closely with that of Twitter users. According to Boschloo’s test^37^, the calculated p-value equaled 1, which exceeds the chosen significance level of *α* = 0*.*27, allowing us to accept the null hypothesis, *H*_0_. This acceptance suggests that the charging sentiment of the BEV in the FG (*p*_2_) aligns with the Twitter charging sentiment (*p*_1_).

In terms of emotions, the Twitter data predominantly showed joy (40%), whereas FGs lacked this joy (Fig. 3c). This could be due to several reasons, including: (a) FG drivers had direct experience with charging, dealing firsthand with challenges like availability and accessibility, while Twitter users may not have had such experience or may have idealised the process, (b) driving campaigns took place in a specific location, in which the charging infrastructure is not well developed, and (c) Twitter users’ positive perception might was influenced by external factors, such as promotional or marketing efforts by charging facility providers. Both groups showed similar interest (anticipation), with FG expressing more negative emotions like sadness and disgust, and comparable anger levels for charging issues.

## Discussion

Despite varying levels of knowledge, both groups exhibited a similar distribution of sentiments towards the two powertrains, with a balance between positive and negative sentiments. However, experts tended to view BEVs more positively, while Twitter users were more favourable towards PHEVs. For the expert group, this difference was explained by the exciting driving experience provided by the instantaneous power delivered by the BEV electric motor, which generated joyous emotions. Compared to the BEV, the PHEV shared annoyances such as the need to charge, but lacked the enjoyable driving traits. In addition, the PHEV was criticised for the need to take care of the maintenance of both the electric and combustion engine, as well as for the limited electric range. The slight tendency of Twitter users to praise PHEVs might have various explanations. First, the fuel flexibility of PHEVs allows users to benefit from both gasoline and electric power, using traditional gas stations, as well as home or public charging stations. This dual capability also helps alleviate range anxiety, offering a longer driving range compared to BEVs. For this reason, the dependency on the availability of charging stations is reduced. In addition, PHEVs, often depicted as the intermediate step of electrification, could be perceived as a more mature technology due to their ability to provide users with the benefits of both gasoline and electric power, enabling users to gradually adapt to electric driving without fully committing to BEVs.

The transportation experts gave a generally positive evaluation of BEVs, which did not translate into a desire to switch to an electric car. Although several participants noted that their opinion towards EVs changed after the driving campaign, the analysis revealed a prevailing feeling of disappointment. This disappointment was attributed to the perceived difficulty-or even impossibility-of switching to an EV due to financial and technical barriers, with the adequacy of charging infrastructure being a major factor.

Charging infrastructure was a recurring topic in both the FG and Twitter discussions/posts, indicating its critical importance. However, different perspectives on the matter emerged. Interestingly, experts only expressed anger in their evaluations when discussing charging, while some Twitter users did so more frequently. This could be indicative of potential detractors expressing harsh criticism of these powertrains, which could introduce bias into the analysis. Interestingly, while anger was absent from the transportation experts’ assessment of the two powertrains, it emerged during discussions about charging issues in a share comparable to that of Twitter users (see Fig. 2c). The combination of negative evaluations and the near absence of positive emotions suggests that experts viewed the charging experience unfavourably. Specifically, drivers identified several weaknesses in both the public and domestic charging infrastructure, perceiving these as significant barriers to EV adoption. In contrast, Twitter users exhibited predominantly positive sentiments towards the charging issue, accompanied by strong emotions such as joy, and relatively limited negative emotions like fear and sadness. Therefore, within the scope of this analysis, charging does not seem to be a major deterrent to EV adoption among the general public.

The disparity in perception may arise from experts having direct experience with EVs, while Twitter users may lack such experience. Additionally, the short duration of the expert driving campaign might have limited their adaptation to EVs, impacting their evaluations. Previous studies^42^ confirm that drivers’ perceptions of EVs improve with practice. In this work, all drivers tested the BEV after gaining experience with the PHEV, resulting in more positive evaluations of the BEV.

The comparison of Twitter users’ and experts’ opinions portrays an interesting picture of the perception of the general public towards alternative vehicles. The two groups of individuals differs for the different understanding of technical aspects of these technologies and usually rely on different sources of information. The knowledge of the general public is often drawn from various informative mass media, such as newspapers, television and online news platform. All these sources, not strictly scientific and peer-reviewed, often expose the public to fake news and false narratives discrediting alternative vehicles^43,44^, potentially affecting consumers’ will to buy one^45^. While experts possess the technical knowledge to debunk mendacious claims and can rely on scientific studies, common people are presumably less prepared to combat disinformation, so that their opinion about these novel technologies would be expected to be more biased. However, the findings of this study seem to controvert this theory. At least for what concerns the sample examined, the opinion of the general public was found to be aligned on several points with that of the experts involved in the experiment. It thus appears that misinformation campaigns carried on by some mass media have produced moderate effects or that, at least, these effects have been mitigated by opposing informative campaigns.

### Actionable recommendations

If public opinion is shaped more by indirect source—which can sometimes spread misinformation or exaggerated claims—the transition to electric mobility could be stalled by consumer hesitation and misplaced expectations. The experts’ more cautious assessment, particularly with regard to charging infrastructure, highlights the need for policies that not only invest in better and more reliable charging facilities, but also ensure that the public is well informed about the real-world performance of BEVs.

Based on our findings, certain actionable policy recommendations emerge:

Recommendations:

① *Coordinated investment in charging infrastructure*: Authorities and institutions should prioritise the deployment of standardised, user-friendly charging networks. Incentives could be provided to accelerate the installation of fast-charging stations and to upgrade existing systems, particularly in regions where the transition to EVs is slower.
② *Consumer education and transparency initiatives*: Future initiatives should support campaigns that disseminate validated, experience-based information on EV performance and maintenance. These initiatives should include testimonials from driving campaigns and independent assessments from trusted experts to counter sensational or misleading messages on social platforms.
③ *Regional and context-specific policies*: An effective policy framework should include localised approaches that take into account regional differences in infrastructure, consumer behaviour and climate. Subsidies or public–private partnerships could be tailored to address specific regional gaps and ensure the accessibility and reliability of charging networks in different environments.
④ *Support transition mechanisms from PHEV to BEV*: Policies could encourage a gradual shift from PHEV models to full electrification by providing trade-in incentives, improved financing for BEVs, and regulatory frameworks that encourage technology upgrades without overburdening consumers.

### Limitations

The analysis, while insightful, has limitations. Firstly, Twitter should not be taken as a comprehensive representation of society, as it may it may skew younger and more digitally engaged^46,47^ under-representing older or less digitally active demographics. Therefore, caution is advised when applying these results across Europe, as regional perceptions vary. Similarly, tweets alone may not reflect general EV perceptions on social media. Other social media platforms, may have different user demographics and engagement patterns that could influence the results. In addition, users who express their views publicly may have responded in a way that they thought was socially desirable, rather than expressing their true opinions, or may have been influenced by the opinions and attitudes of others. Also, the data may be skewed by individuals defending specific interests. Although commercial and political profiles were excluded, some *influencers* and *detractors* expressing extreme opinions, could remain. The data collection period may also have been affected by specific events or announcements related to EVs, such as incidents, government incentives, or new product launches. These events could have influenced public perceptions and opinions, limiting the generalisability of the findings. Moreover, a possible bias arises as social media participants may lack EV experience, unlike participating experts who directly engaged with the technologies. Lastly, the authors note that while the sample size of 10 experts was sufficient for the purposes of the FGs reaching code saturation already from the second FG as very few sub-codes arose, it may not be fully representative of the broader population of transportation experts.

The data from the transportation experts, despite being a small group, achieved 80% topic saturation with just two to three FGs indicating that the FG methodology was effective^48,49^. Although diverse in scientific expertise, this sample may not fully represent the broader population of transportation experts. The comparison with a larger social media sample adds significant value. The study’s focus on northern Italy might have influenced perceptions due to local conditions. Moreover, care should be taken when generalising the results of the driving campaign to the wide and diverse market of plug-in and electric vehicles, since all the models driven by the participants belonged to the same car segment, C.

Future research should bridge these gaps. Expanding to other social media platforms can help to identify common trends and concerns regarding EVs, as well as facilitate cross-platform sentiment analysis. Secondly, investigating national particularities and stages of EV market maturity through case studies in specific European countries. This will help develop context-specific policy recommendations. Additionally, examining other topics within EV discussions, such as incentives, can help identify main barriers and assess whether they vary across different user groups, such as urban versus rural residents or users with varying income and technology access.

## Methods

The study examined EV adoption through two lenses: the views of transportation specialists and the opinions of the general public. To mitigate cognitive biases against vehicle powertrain technology^50,51^ a specialised driving campaign was conducted. Ten drivers participated, operating three vehicles, starting with the ICE model and progressing first with the PHEV and then with the BEV. The ICE vehicle served as benchmark of the leading powertrain technology in the EU market. In order to favour the comparability of the three vehicles, all the models used for the driving campaign were chosen from the same market segment. Specifically, the all belonged to the C segment of the Eurocar classification and, thus, shared comparable technical specifications and sale prices. This choice was made to mitigate possible interferences in the perception of the three vehicles unrelated to the type of powertrain.

**Fig. 4:**
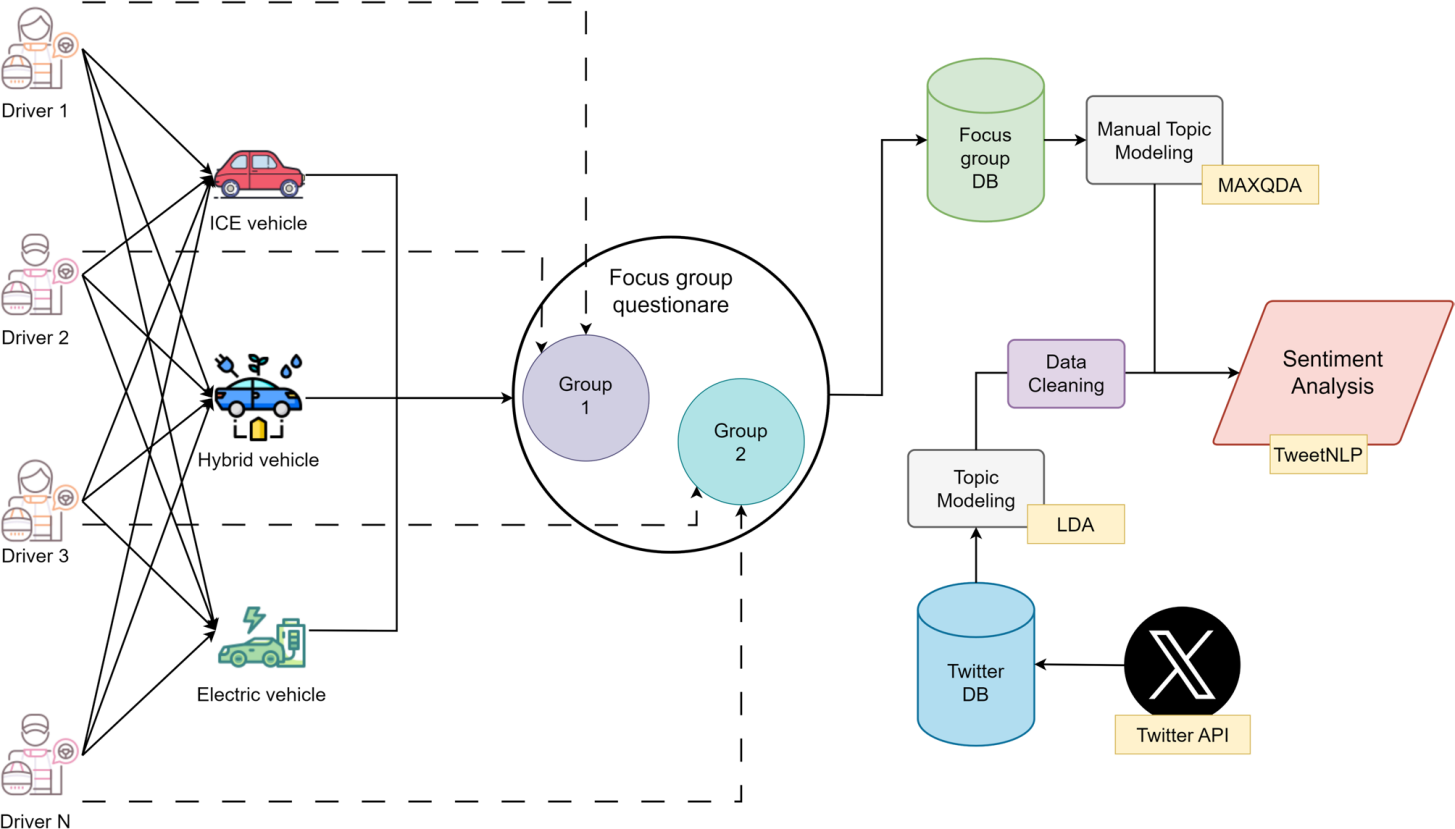
Methodology overview. The initial segment of the methodology delineates the textual campaign, during which a number of drivers operated three distinct vehicles and participated in a FG discussion. The right-hand portion of the diagram elucidates the procedures implemented for data processing and analysis.

Participants were required to drive each of the three designated vehicle types for a duration of 2–4 weeks each. All the drivers drove the same vehicle types (see Fig. 4).

Following the driving phase, structured FG discussions were held with two driver subgroups. A standardised questionnaire was used to guide the discussions, which covered topics such as vehicle assessment, charging experience, and opinion shifts on powertrain technologies (central part of Fig. 4).

Simultaneously, EVs tweets were collected in all EU countries from June 2019 to June 2024, aligning with the time frame of the driving campaign. Sentiment analysis was performed on the collected tweets and the responses from the FGs. Finally, the sentiments towards the EVs expressed in the FGs were then compared with those observed on social media, drawing insights based on the results of the sentiment analysis.

### Focus group methodology

Transport experts were contacted and invited to participate in a real-life driving campaign. During the recruitment process, the research team engaged in direct communication with potential participants, informing about the study’s objectives and aims through face-to-face conversations. Following a comprehensive screening, ten experts were selected for achieving theoretical saturation^52,53^ and were divided into two homogeneous groups of 5 participants^54^. All affiliated with a research centre in northern Italy, were selected to drive the three distinct vehicles. Participants were provided with the Targeted Consultation Activities Privacy Statement (Record Reference: DPR-EC-01011). Informed consent was obtained from all participants, and, when necessary, from their legal guardians, ensuring that everyone was fully aware of the study’s nature and scope. The selected experts had specialised expertise in fields such as civil engineering, environmental science, transportation economics, data science, mechanical engineering, urban planning, policy, and information technology. Subsequently, they were invited to participate in audio–video recorded FGs, each lasting two hours, to discuss their experiences.

FGs were selected as the research method for this study due to their effectiveness in exploring participants’ experiences and attitudes through interactive discussions, fostering dynamic group interactions^55^. This qualitative approach is widely used to gather insights on scientific advancements, promote collaborative governance^56^, and is particularly common in mobility and transportation research as evidenced by many studies^21,57–60^. In this study, FGs facilitated an interactive and comprehensive exploration of participants’ driving experiences, as outlined by Lune and Berg^61^.

Participants were informed about the recording and transcription process and provided consent for the data to be used solely for research purposes. Each FG session was two hours long, led by a moderator with a co-moderator observing. All recordings were anonymised, transcribed verbatim, and names were replaced by pseudonyms to ensure privacy. The sessions were structured using a standardised guide created by the research team to ensure consistency across all sessions, covering three main sections:

1. *Introduction*: Purpose of the discussion, housekeeping rules, audio-recording registration, and an icebreaker activity.
2. *General evaluation*: Advantages and disadvantages of the driven vehicles, trip purpose, observed differences among commuting purposes, driver input, safety feelings, charging experiences, and environmental impacts.
3. *Willingness to buy/change vehicle*: Cost comparisons, campaign effect on opinions, EV subsidies, and other reasons for purchasing an EV.

The transcripts were then imported into the MAXQDA qualitative data analysis software (VERBI Software, MAXQDA, version 2020) for comprehensive categorisation and coding. A combined inductive and deductive approach was used, applying top-level codes to all transcripts. The research team used a custom coding system for specific segments, while additional codes were identified inductively during the analysis process. Inter-coder reliability testing was implemented to maintain consistency and reliability throughout the coding process.

All methods were executed in strict adherence to the relevant guidelines and regulations to ensure the integrity and ethical standards of the research. The experimental protocols implemented in this study received approval from the Research Ethic Board of the Joint Research Centre of the European Commission ensuring compliance with institutional ethical standards. According to the ethical standards, a pseudoanonymisation was applied to all the names in this manuscript.

### Categorisation of FG excerpts and dataset composition

Up to four hierarchical code levels were assigned to the excerpts derived from the transcript. The first code level concerns the main topic of the discussion. Table 1 below shows the number of excerpts collected for each discussion topic. The comprehensive code explanation can be found in Table 2, located in Appendix B. A total of 357 excerpts were collected and classified into 11 topics, ranging from a *General evaluation* of the experience with the three cars to more specific themes relevant to EVs adoption, such as *Charging experiences*, *Expenditure comparison*, *Environmental perspectives*, etc.

**Table 1:** Classification of excerpts from the FG transcript according to discussion topics. The table shows the number of excerpts classified according to the discussion topics defined during the analysis of the transcript. N.B., introductory talks, which were excluded from the analysis, are not reported.

Code 1—Topic	Number of excerpts
General evaluation	132
Charging experiences	85
Differences in driving	38
Change of opinion	25
Expenditure comparison	22
Other reasons for changing a car	13
Other advantages and disadvantages of EVs	12
Incentives subsidies	10
Environmental perspectives	9
Ideal situation for buying a BEV	6
Actions in parallel with EV deployment	5
Total	357

**Table 2:** Classification of excerpts from the FG transcript according to discussion topics and car types. The table shows the number of excerpts classified according to the car they refer to for each discussion topic.

Code 1—Topic	Code 2—Car		
	*ICE*	*PHEV*	*EV*
General evaluation	28	50	54
Charging experiences	0	16	15
Differences in driving	11	14	3
Change of opinion	0	2	1
Expenditure comparison	5	6	7
Other reasons for changing a car	1	1	4

Whenever possible, a second code level was assigned to specify the car each excerpt refers to. This was not always feasible as some excerpts referenced multiple cars. Table 2 below shows the number of excerpts available for each car and discussion topic. The other sub-codes were assigned to refine the classification of the discussion topic, and are therefore strongly related to it.

### Twitter queries dataset

Twitter is a social media platform that allows users to post and interact with messages known as ’tweets’. These tweets can be up to 280 characters long and can include text, images, videos, and links. With regard to interaction, users can follow other accounts, retweet, like, or reply to tweets they find interesting or important.

The dataset for this research was collected via the Enterprise API, formerly the ”GNIP 2.0 API,” and also known as the academic research track of the Twitter API. This platform provides access to a comprehensive archive of historical public tweets from July 2006 onwards. A recent study by Pfeffer et al.^62^ evaluates the thoroughness and temporal consistency of the data acquired through Twitter’s academic API. Their research indicates that the search outcomes are reliable and yield (almost) exhaustive samples of Twitter data encompassing a broad range of search queries.

In this study, a set of keywords consistent with the terminology used in previous studies were used^12,13^. The keywords include: ”electric vehicle”, ”EV”, ”charging station”, ”electric car”, ”PHEV”, ”plug-in hybrid”, and ”plug-in EV”. In addition, specific terms such as ”EV charging”, ”electric vehicle charging”, and ”charging station” were added to define charging events more precisely. For PHEV-related queries, terms such as ”plug-in hybrid electric vehicles”, ”PHEV”, ”hybrid cars”, and ”hybrid” were incorporated. Beyond the main content, the collection includes various features supported by the Twitter API. This includes the geographic location of the tweets, details related to the users, and other relevant aspects. Data collection is selective, concentrating solely on tweets generated in EU countries. The data was gathered using a unique pairing of country and language. For each country, we translated the queries into the official language.

**Fig. 5:**
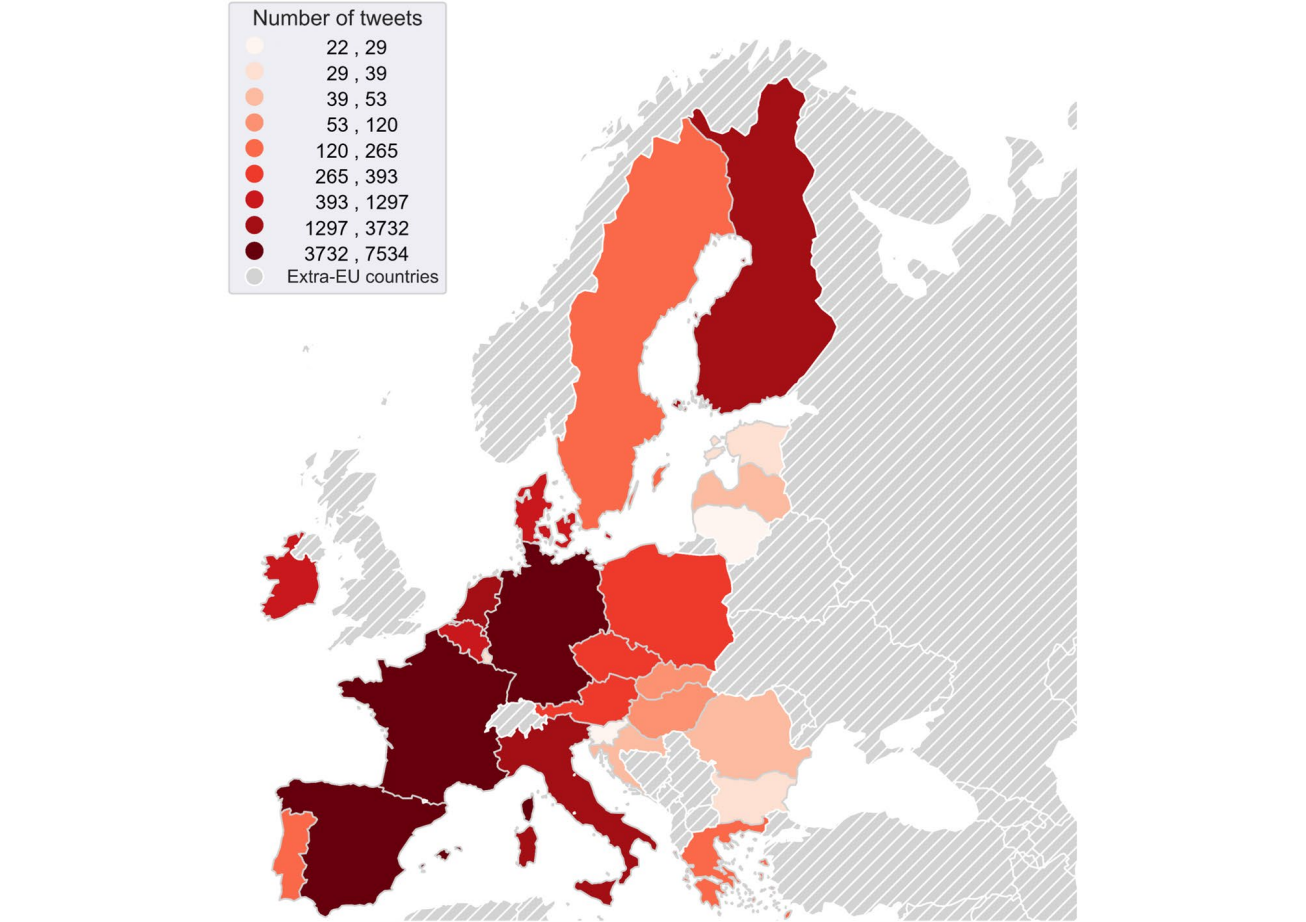
Distribution of tweets in the cleaned dataset according to the country of origin. The colour density represents the number of tweets. Extra-EU countries are hatched in grey.

The original dataset comprised 30,710 tweets collected from June 2019 to June 2024. Of these, 866 tweets were removed because they were predominantly composed of non-textual elements, including hashtags, media links, and mentions. Additionally, tweets from verified business and government accounts were removed to mitigate the potential influence of industrial interests and political biases. This resulted in the exclusion of another 970 tweets. Consequently, the refined dataset comprises 28,874 entries for analysis (Fig. 5).

To discern the various subjects linked to the tweets, they were classified through topic modelling utilising the Latent Dirichlet Allocation (LDA) algorithm^63^. In this study, the LDA was performed using the Mallet tool incorporated into the *Gensim* Python library^64^. The LDA process produces two distinct matrices: the initial matrix displays the distribution of topic probabilities between documents and is formatted as an *N* × *K* matrix; the second matrix indicates the distribution of word probabilities across topics, formatted as a *K* × *V* matrix. Here, *N* represents the total number of tweets, *K* designates the count of distinct topics, and *V* corresponds to the vocabulary size, that is, the total number of unique terms present in the entire collection of texts. To determine the ideal number of topics, one may refer to the coherence score, a metric that evaluates the quality of the topic modelling outcomes. A greater coherence score suggests a higher degree of logical consistency within the results of the topic modelling^65^.

**Fig. 6:**
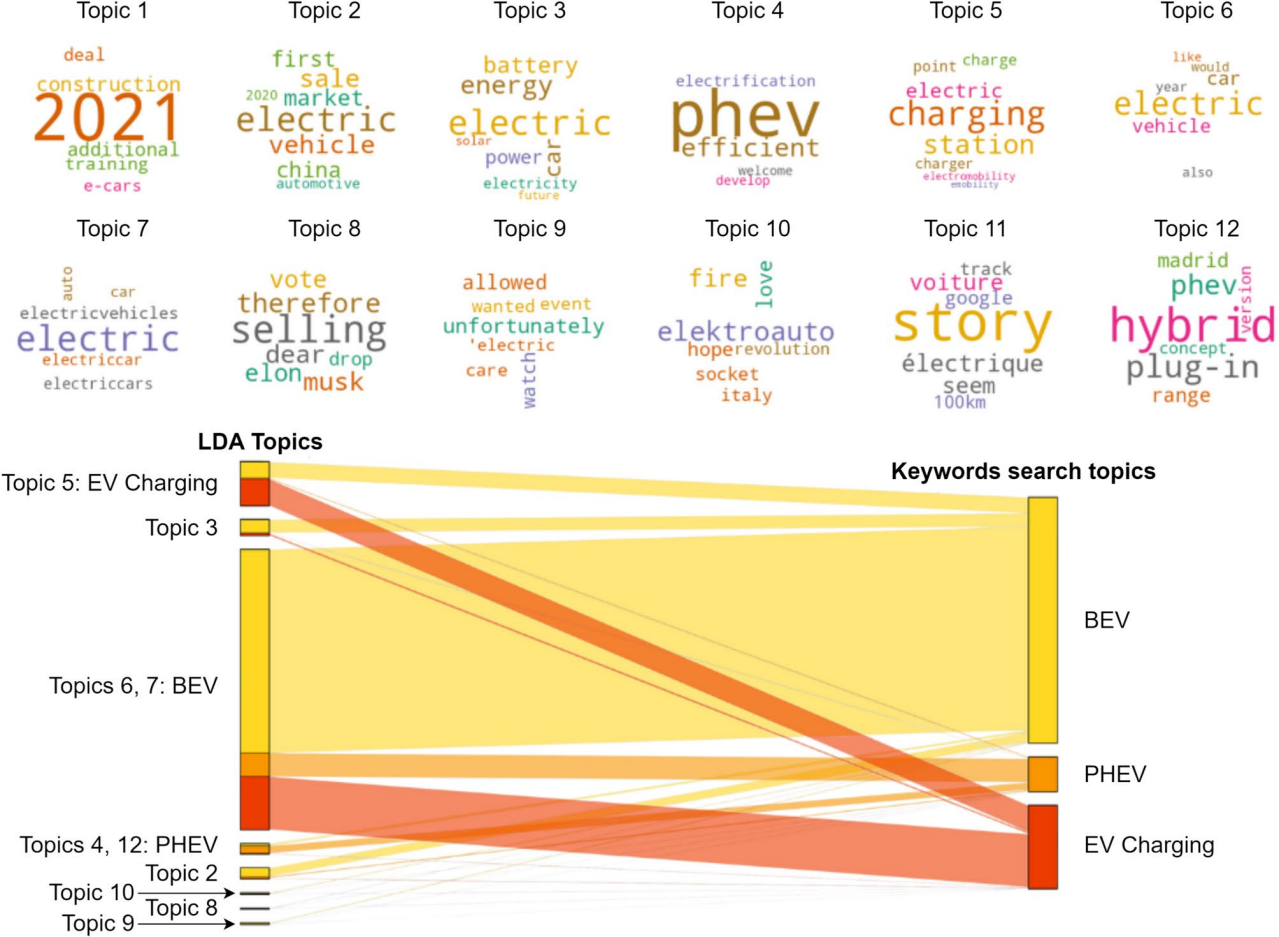
Topics identified using the LDA algorithm and their relation to keyword searches. Word clouds illustrating the topic modelling results (Number of topics = 12). Overlap between topics identified via LDA (left) and through keyword searches (right). The width of the flows between both sides indicates the number of tweets involved. Flows that connect matching topics on both sides represent overlapping results from both methods. Diverging flows to different topics signify that the tweets were categorised into separate topics by the methods. Note: Topics 1 and 11, deemed marginally relevant to this analysis, were excluded.

This research indicates that selecting between 12 to 14 topics results in high coherence scores and yields the clearest and most comprehensive thematic distinctions; the full methodology applied can be found in the Appendix B. Following a thorough manual evaluation, as suggested by Grimmer et al.^66^, twelve were determined as the optimal number of topics for this analysis. The most frequently occurring words within each topic following the application of topic modelling and their relation to keyword search are represented in Fig. 6. Topic 1 and Topic 11 were considered non-essential for the purpose of this study. Topic 1 consists of tweets discussing construction topics, Fig. 6, that is, a topic pertaining to electric vehicle production, which are not pertinent to the objectives of this paper. Meanwhile, Topic 11 comprised tweets with inadequate translations, often due to the presence of abbreviated words or slang. As the emotional analysis focuses solely on English-language content, tweets related to Topic 11 were excluded.

Upon excluding Topics 1 and 11, the dataset is reduced to comprise 28,655 tweets. The study compares expert and public perspectives on BEV, PHEV, and EV charging. Tweets were reclassified into these three categories using a keyword search (Fig. 6).

To ensure the representation of all EU countries within the dataset, the hashtags and search terms previously mentioned have been translated into the official languages of each country. Consequently, for every country, the search is conducted in both English and the respective official languages in order to maximise the collection of relevant tweets. The decision to allow multiple languages in the search criteria has resulted in a data set where tweets are evenly distributed among EU member states, as depicted in Fig. 5. This distribution generally reflects the population size of the respective countries. However, there are some significant deviations from this pattern. For instance, Poland, which ranks as the fifth most populous EU nation, is underrepresented in the dataset. These disparities are likely attributable to the varying levels of popularity of the subject matter, as discussed in the referenced literature^67^. Despite these minor discrepancies, the dataset is considered sufficiently representative for the purpose of analysing the EU’s perception of EVs.

### Sentiment analysis model

We leverage the TweetNLP^68^ platform for the analysis of sentiments and emotions in tweets. TweetNLP is grounded in Transformer-based language models^68^ pre-trained on Twitter datasets, differentiating it from traditional NLP models, which are often trained on more formal and polished texts such as news articles or Wikipedia.

TweetNLP incorporates several language models to enhance its analytical capabilities. The TweetEval model, based on RoBERTa^69^, has been initially pre-trained on a large corpus of 60 million tweets. Building upon this, the TimeLMs model^70^ extends the capabilities of TweetEval by training on an even more substantial and continuously updated collection of tweets, which currently numbers over 132 million. Additionally, the XLM-T model^71^ brings multilingual analysis into the fold, having been trained on a massive dataset that includes 198 million tweets spanning more than 30 languages. These models collectively ensure that TweetNLP is adept at handling the multifaceted nature of social media communication. Overall, the choice of TweetNLP was driven by two factors: (1) Exclusive training on Twitter data, allowing it to effectively process both formal and informal text relevant to various contexts, including FGs and Twitter datasets; (2) A robust framework optimised for sentiment analysis, based on the RoBERTa architecture, an enhanced version of BERT^72^.

TweetNLP was used to estimate the likelihood of each tweet’s sentiment being positive, negative, or neutral, ensuring the total probability sums to 1. For emotion analysis, the probability of each tweet was calculated, conveying emotions such as anger, anticipation, disgust, fear, joy, love, optimism, pessimism, sadness, surprise, trust, or no emotion, with the total probability also summing to 1.

Tweets in languages other than English, French, German, Italian, Portuguese, and Spanish were translated into English for uniform processing with TweetNLP. All tweets were translated into English for emotion analysis, as it is the only language supported by the tool for this purpose.

### Statistical analysis

When the data sample is small, the Fisher test^73^ is commonly employed as the standard statistical test^74^. Under these circumstances, Boschloo’s test^37^, which uses the p-value from the Fisher test as its test statistic in an exact unconditional test, proves to be consistently more powerful than the Fisher test and is similarly recommended^75^. In the current work Boschloo’s test was employed to assess whether there are statistically significant differences in sentiments about various vehicles. Known for its precision, this test is an exact method used for analysing contingency tables^76^. It examines the link between positive and negative sentiments. The choice of Boschloo’s test is justified by its uniformly greater power compared to Fisher’s exact test for 2 × 2 contingency tables^76^. The test was executed using the Scipy Python library^77^. When we use Boschlo’s test, we can assert three different alternative hypotheses:

- If alternative is less: The null hypothesis is H_0_: p_1_ = p_2_ with alternative hypothesis H_1_: p_1_ < p_2_
- If alternative is greater: The null hypothesis is H_0_: p_1_ = p_2_ with alternative hypothesis H_1_: p_1_ > p_2_
- If alternative is two-sided: The null hypothesis is H_0_: p_1_ = p_2_ with alternative hypothesis H_1_: p_1_≠ p_2_

Initially, we conduct a statistical analysis within the FG. Given that the FG campaign utilized ICE as a benchmark vehicle, we begin by comparing whether the positive and negative sentiment for PHEV and BEV differs when assessed against the ICE vehicle. Lastly, we also examine any variances in sentiment between the PHEV and BEV themselves. A similar analysis was conducted for the Twitter dataset to determine if there were any statistical disparities in the perception of PHEVs compared to BEVs.

The FG statistical analysis was divided into three parts: the general evaluation, the charging experience, and shifts in opinion. Due to the varying number of responses in each group, distinct confidence intervals or significance level were employed to minimize Type I and Type II errors in Boschloo’s test^78^. The α value was determined using their official R code (https://github.com/Lakens/justify_alpha_in_practice).

Regarding general evaluation of the vehicles, charging experience, and change of opinion, the α values were derived as 0.22, 0.27, and 0.27, respectively in the FG. In terms of the statistical analysis conducted on the Twitter data, the α value stood at 0.05, given that the minimum count of comments consistently exceeded^64^. Lastly, when comparing the FGs dataset with the Twitter dataset, the α value was 0.22 for the sentiment analysis between PHEV and BEV, whereas it was 0.27 for the charging experience evaluation.

## Supplementary Information

Supplementary Information.

## Data Availability

The datasets generated and/or analysed during the current study are available from the corresponding author (georgios.fontaras@ec.europa.eu) upon reasonable request.
