# Supplementary material for: Comparative analysis of public and expert perceptions of electrified vehicles in the European Union
Source: Sci Rep. 2025 Jul 1;15:21695. doi: 10.1038/s41598-025-06071-0 (PMC12214769; doi:10.1038/s41598-025-06071-0)
Supplement: Supplementary file 1 — Supplementary Information. [file 41598_2025_6071_MOESM1_ESM.docx]

# Appendices

## Appendix A: Focus group impressions

The BEV impressed participants with its silent and comfortable driving experience, free from engine noise and vibrations. Instant power and electronic driver assist further enhanced the overall driving experience. Regarding engine acceleration, the BEV was described as more responsive and agile, especially when driving uphill and quick turns, while the ICE felt less prepared and showed comparatively weaker performance in these scenarios. However, some participants noted that fast acceleration required an adjustment in responsiveness. This double-edge vision of the BEV’s driving performance is well summed up by the following quote:

*It could become a threat because if anybody starts enjoying battery electric vehicles so much, they will all accelerate super-fast from traffic lights and stops.* (Edoardo)

Participants also found the behaviour of the BEV more difficult to predict than the PHEV electric mode. The BEV showed an unexpected delay in acceleration after a hard deceleration. Inconsistent braking behaviour was mentioned twice, particularly in relation to varying battery charge levels, and was considered susceptible to be a significant safety concerns.

Other concerns were raised about the reliance on charging infrastructure, with access to charging stations and the vehicle’s range varying by location, potentially causing anxiety during long trips or in areas with limited charging facilities. Participants also raised concerns about range anxiety and how different features, such as air conditioning, could affect the driving range, with the advertised range potentially being influenced by driving conditions. The contrast between the exciting driving experience provided by the BEV and the incumbent range anxiety is well summed up by the following excerpt:

*It is very fun, really, really very fun, going up the hills is amazing with the handling and everything with an electric car. However, it is the moment where all the weight of the electric car with the battery can be seen, so you need to recharge. You probably need to recharge when you are up, not when you go down.* (Pietro)

## Appendix B: Focus Group discussion script and execution

Participants were selected through personal contacts and were all affiliated with the same research center in northern Italy. They were contacted via email, with the only requirement being a valid driving license, ensuring that their expertise spanned a wide range of transportation topics. To maintain objectivity, no pre-discussion materials were provided, and participants were unaware of the specific topics to be discussed, both before and after driving the vehicles, as well as during the FGs.The script used during the FGs was identical for both sessions, with questions drawn from existing studies in the literature. The FG was structured to begin with an introductory “warm-up” section, followed by specific aspects related to the three powertrains driven, and then more general topics, such as subsidies awareness and cost considerations. The questionnaire’s structure was chosen following the standardized method outlined by Breen[1](#_bookmark5). This original structure was modified to enabled the capture of both emotional reactions and practical considerations while facilitating comparisons and contrasts between the three technologies. The FGs were semi-structured, with participants asked predefined questions but also allowed to introduce additional topics.

Two in-person FGs were conducted, each with 5 participants, held in a meeting room at the organization. Each session lasted 2 hours, with 30 minutes allocated to the first section and 45 minutes for each of the following two sections. Table [1](#_bookmark1) provides more information. During the FGs, a moderator led the conversation, while a co-moderator observed. The moderator’s role was to pose predefined open-ended questions (prepared by the research team) and encourage full participation from all members. The question flow began with broad, open-ended inquiries and gradually became more specific based on participants’ feedback. For instance, we started with general questions about the overall charging experience and then moved to more focused topics such as charger compatibility and the use of charging applications.

To ensure a natural, dynamic discussion, participants were encouraged to interrupt each other, allowing contributions at the right moment rather than waiting until the end of someone’s speech, which could diminish relevance. The dominance of more vocal participants was managed by actively encouraging others to speak.

In order to ensure data triangulation, two researchers were involved in the coding process using MAXQDA software to validate findings and minimise potential biases in interpretation. The researchers independently coded the same data, and their results were then compared for consistency or any discrepancies. Additionally, we used MAXQDA’s visualisation tools, such as code matrices, to identify patterns and inconsistencies across data sources.

For the analysis of the FGs, we employed both deductive and inductive coding approaches, see Table [2](#_bookmark2). Initially, a deductive approach was used with a top-down coding strategy, applying pre-defined “parent” codes based on the sections of the script used during the discussions. These primary codes aligned with the script sections and were selected based on existing literature, focusing on themes frequently discussed in prior research. Certain topics were also included based on the researchers’ specific interests, such as the “expenditure comparison.” As the analysis progressed, we adopted an inductive approach, refining the first-level codes into more specific subcodes. New subcodes emerged directly from the transcriptions, and some codes were merged or split based on patterns that emerged during the data analysis.

## Appendix C: Data collection queries

In this study, the compilation of queries was derived from comparable research on BEVs[2](#_bookmark6), [3](#_bookmark7). As our analysis covered all EU countries, we translated the proposed queries into the official languages of each respective nation. A query structure is defined below:

*{*

” query ” : ” ( e l e c t r i c v e h i c l e OR c h a r g i n g s t a t i o n OR e l e c t r i c c a r OR phev OR plug − i n h y b r i d OR plug − i n ev ) ”

” p l a c e c o u n t r y ” : ” ISO c o u n t r y code ” ” l a n g ” : ” Language code ” ,

” I n i t i a l d a t e ” : ” 2 0 1 9 / 0 1 / 0 1 0 0 : 0 0 ” ,

” F i n a l Date ” : ” 2 0 2 4 / 0 5 / 3 0 1 2 : 0 0 ”

*}*

The combination of country and languages can be found in Table [3](#_bookmark3). The translation of the queries were done using the DeepL API.

## Appendix D: Number of topics identified

The quantity of topics was chosen to reduce text overlap and enhance coherence, specifically optimising the separation between distinct topics in the latent space produced by the LDA algorithm. The optimal number identified is shown in Figure [1](#_bookmark0).


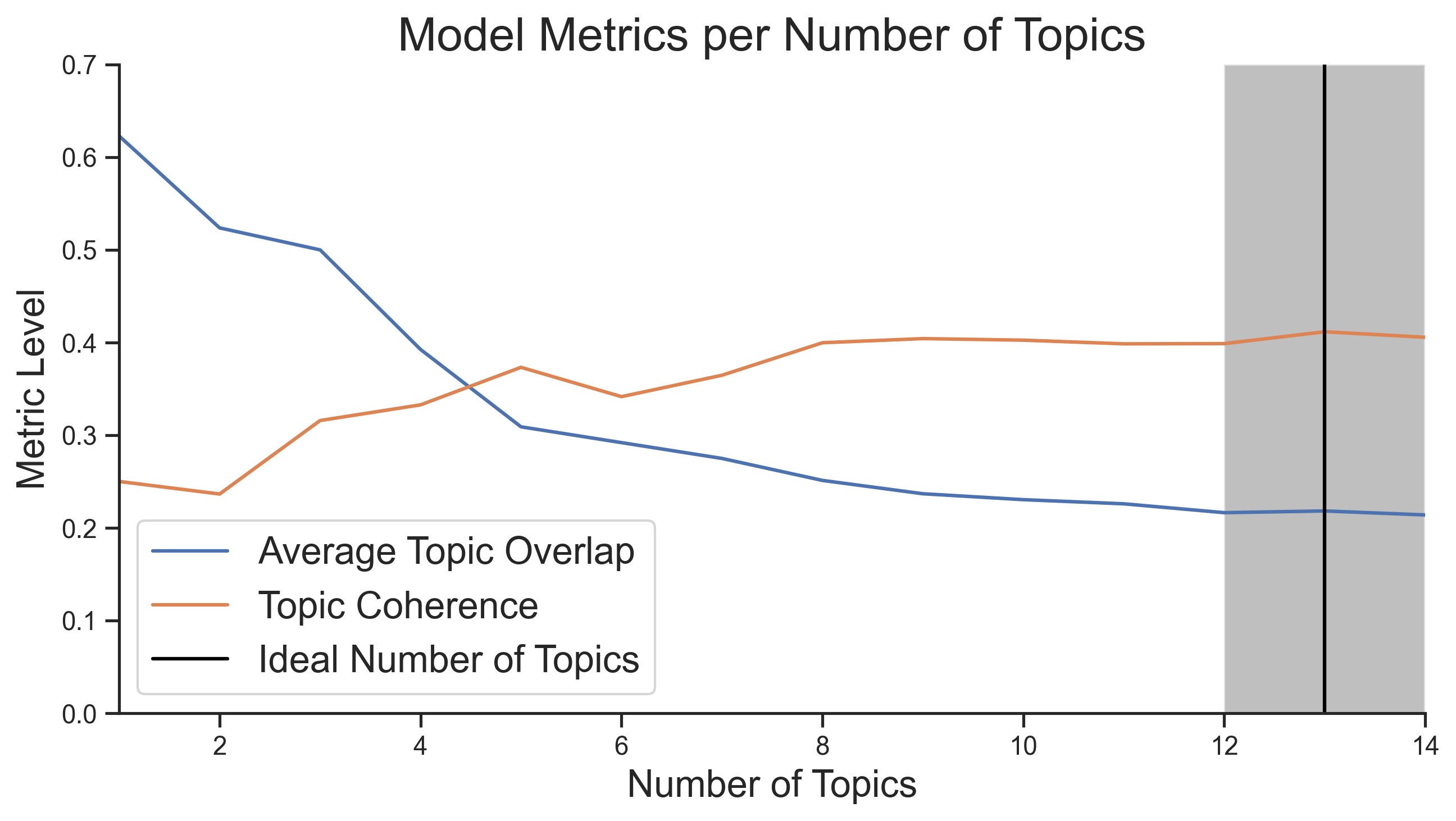


**Figure 1. Topic selection model metrics per number of topics.** The orange line shows the coherence of each topic. The blue line shows the average overlapping between topics.

Section Questions Introduction

- Welcome
- Presentation of moderators, role
- Purpose of the discussion
- Housekeeping rules
- Brief presentation of participants (name, age, motivation of participating to the driving cam- paigns)
- Ice-breaking activity

General Evaluation

- Reflect on your experience with each vehicle and summarise it using one word per vehicle, then elaborate on these words.
- How was your experience with the three vehicles you drove? What are the main advantages and disadvantages?
- Did anything make you happy or annoyed while driving these cars?
- Did you use them mainly for commuting or leisure? If both, did you notice any differences between commuting and leisure?

Operational aspects

- Did you notice any differences in engine power among the three vehicles? Did any of these vehicles respond faster to your inputs?
- Do you feel that you drove the PHEV differently compared to the conventional vehicle? Could you please explain your answer?
- Could you describe your charging experience? Where did you charge the vehicle? Did you have any charging facilities at home? Did you encounter any issues with the charger’s compatibility? Did you need an adapter? Was the process easy? What about the cost?

Environmental aspects

- From an environmental perspective, what are your thoughts on these three vehicles? What are their respective impacts on the environment?

Willingness to

buy/change vehicle • What type of vehicle do you own?

- - Did you compare the costs among the vehicles you drove? If so, what were the results?
  - Has your experience with this campaign made you consider changing your vehicle? If yes, which type of vehicle are you considering?
  - Are you aware of EV subsidies? Which ones do you know of that are currently available in Italy? Would these incentives encourage you to buy an EV?
  - Are there any other factors that would make you consider purchasing a vehicle with a different technology?

**Table 1.** The table outlines the structure designed for conducting the FG discussion.

## Code 1 - Topic Description

General Evaluation This code collects feedback on general impressions of the vehicles, in- cluding likes and dislikes. It gathers a one-word summary of the experi- ence with each vehicle, along with details on advantages, disadvantages, and any emotional reactions. It also explores usage (commuting vs. leisure), differences in engine power or responsiveness, and whether the PHEV was driven differently from the conventional vehicle.

Charging Experiences This code focuses on charging experiences, collecting information on where and how the vehicles were charged, the availability of home charging facilities, any issues with charger compatibility or the need for adaptors, the ease of the process, and the cost.

Other Reasons for Changing a Car This code includes factors such as status or income that could influence

the decision to consider purchasing a vehicle with a different technology.

Differences in Driving This code collects information on whether the PHEV was driven dif- ferently from the conventional vehicle and the BEV, along with an explanation for the response.

Change of Opinion This code collects information on whether the experience with the cam- paign influenced the decision to consider changing the vehicle, and if so, which type of vehicle would be considered.

Other Advantages and Disadvantages of EVs This code includes additional advantages and disadvantages of EVs that

were not directly experienced by the participants during the campaign, but are based on their experiences with other vehicles or information they have heard.

Other Advantages and Disadvantages of EVs This code includes additional advantages and disadvantages of EVs that

were not directly experienced by the participants during the campaign, but are based on their experiences with other vehicles or information they have heard.

Environmental Perspectives This code collects an evaluation of the environmental impact of the three

vehicles.

Expenditure Comparison This code collects information on whether a comparison was made

regarding the expenditures associated with the three vehicles driven, and if so, what the result was.

Incentives/Subsidies This code collects information on the participant’s awareness of EV subsidies, which ones are currently available in Italy, and whether these subsidies would influence the decision to purchase an EV.

Actions in Parallel with EV Deployment This code includes initiatives or measures suggested by participants that

should be taken alongside the deployment of electric vehicles (EVs) to support their successful integration into the market.

Ideal Situation for Buying a BEV This code includes all the elements, according to participants, that would

constitute an ideal situation for purchasing a BEV.

**Table 2.** Code followed during the FGs answers analysis.

## Country Lenguages

Austria German Belgium Dutch, French, German Bulgaria Bulgarian

Croatia Croatian Cyprus Greek, turkish

Czech Republic Czech Denmark Danish

Estonia Estonian Finland Finnish, Swedish France French

Germany German

Greece Greek

Hungary Hungarian

Ireland English

Italy Italian

Latvia Latvian

Lithuania Baltic Luxembourg German, French

Malta English

Netherlands Dutch Poland Polish

Portugal Portugish

Romania Romanian

Slovakia Slovak

Slovenia Slovenian

Spain Spanish

Sweden Swedish

**Table 3.** Combinations of countries and languages applied in the translation of queries.

# References

1. Breen, R. L. A practical guide to focus-group research. *J. geography higher education* **30**, 463–475 (2006).
2. Ruan, T. & Lv, Q. Public perception of electric vehicles on reddit and twitter: A cross-platform analysis. *Transp. Res.* *Interdiscip. Perspectives* **21**, 100872, DOI: <https://doi.org/10.1016/j.trip.2023.100872> (2023).
3. Ruan, T. & Lv, Q. Public perception of electric vehicles on reddit over the past decade. *Commun. Transp. Res.* **2**, 100070, DOI: <https://doi.org/10.1016/j.commtr.2022.100070> (2022).
